# Simulating *in vitro *transcriptional response of zinc homeostasis system in *Escherichia coli*

**DOI:** 10.1186/1752-0509-2-89

**Published:** 2008-10-24

**Authors:** Jiangjun Cui, Jaap A Kaandorp, Catherine M Lloyd

**Affiliations:** 1 Section Computational Science, Faculty of Science, University of Amsterdam, Kruislaan 403, 1098 SJ Amsterdam, The Netherlands; 2 Bioengineering Institute, University of Auckland, Level 6, 70 Symonds Street, Auckland, New Zealand

## Abstract

**Background:**

The zinc homeostasis system in *Escherichia coli *is one of the most intensively studied prokaryotic zinc homeostasis systems. Its underlying regulatory machine consists of repression on zinc influx through ZnuABC by Zur (Zn^2+ ^uptake regulator) and activation on zinc efflux via ZntA by ZntR (a zinc-responsive regulator). Although these transcriptional regulations seem to be well characterized, and there is an abundance of detailed *in vitro *experimental data available, as yet there is no mathematical model to help interpret these data. To our knowledge, the work described here is the first attempt to use a mathematical model to simulate these regulatory relations and to help explain the *in vitro *experimental data.

**Results:**

We develop a unified mathematical model consisting of 14 reactions to simulate the *in vitro *transcriptional response of the zinc homeostasis system in *E. coli*. Firstly, we simulate the *in vitro *Zur-DNA interaction by using two of these reactions, which are expressed as 4 ordinary differential equations (ODEs). By imposing the conservation restraints and solving the relevant steady state equations, we find that the simulated sigmoidal curve matches the corresponding experimental data. Secondly, by numerically solving the ODEs for simulating the Zur and ZntR run-off transcription experiments, and depicting the simulated concentrations of *zntA *and *znuC *transcripts as a function of free zinc concentration, we find that the simulated curves fit the corresponding *in vitro *experimental data. Moreover, we also perform simulations, after taking into consideration the competitive effects of ZntR with the zinc buffer, and depict the simulated concentration of *zntA *transcripts as a function of the total ZntR concentration, both in the presence and absence of Zn(II). The obtained simulation results are in general agreement with the corresponding experimental data.

**Conclusion:**

Simulation results show that our model can quantitatively reproduce the results of several of the *in vitro *experiments conducted by Outten CE and her colleagues. Our model provides a detailed insight into the dynamics of the regulatory system and also provides a general framework for simulating *in vitro *metal-binding and transcription experiments and interpreting the relevant experimental data.

## Background

Zinc is essential for life. It serves as a structural or catalytic cofactor in a large number of proteins such as RNA polymerase and zinc finger proteins [1-9]. Zinc also plays an important signalling role in various biological processes such as neurotransmission, cell proliferation, and apoptosis [10,11]. However, due to the potential toxicity of zinc, intracellular zinc concentrations must be kept under tight control. For example, a high intracellular Zn^2+ ^concentration can inhibit the aerobic respiratory chain in *E. coli *[6-8].

**Figure 1:**
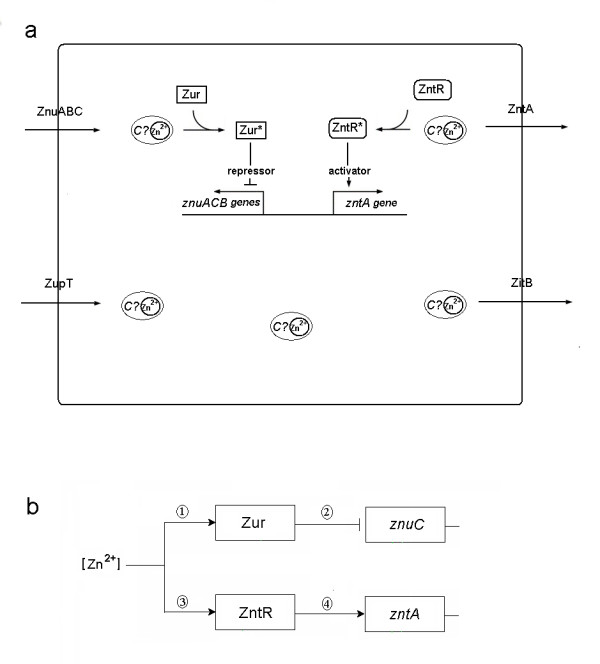
**Schematic representations of *E. coli *zinc homeostasis system and the *in vitro *sub-processes**. (a) A schematic graph depicts the Zn^2+ ^homeostasis system in *Escherichia coli*. Extracellular Zn^2+ ^enters the cytoplasm through ZnuABC and ZupT [7,19]. In the presence of zinc, Zur binds to the *znu *operator and represses the transcription of *znuACB *gene cluster [8,20]. Excess intracellular zinc ions are exported by ZntA and ZitB [16,17,21]. Intracellular zinc can bind with protein ZntR and convert it into a strong transcriptional activator of the *zntA *gene [8,14,29]. The cytoplasmic zinc trafficking may involve chaperone-like proteins [22]. Abbreviations used in this graph are as follows: Zur* (active Zur); ZntR* (active ZntR); C? (zinc chaperone whose existence is still under debate) [2,22]. (b) A schematic graph depicts the main sub-processes which we need to model for simulating *in vitro *transcriptional response: (i) Zn^2+^-sensing by Zur, (ii) Transcriptional repression of *znuC *gene by Zur, (iii) Zn^2+^-sensing by ZntR and (iv) Transcriptional activation of *zntA *gene by ZntR (Please note that here we only model the transcription of *znuC *gene rather than of the whole *znuACB *gene cluster because we only have reported data for *znuC *transcripts available for comparison) [22].

*E. coli *achieves zinc homeostasis by regulating the uptake and efflux of zinc across the plasma membrane [1,8]. As we can see in Fig. 1a, extracellular zinc ions are transported into the cytoplasm through ZnuABC (an ABC-type transporter) and ZupT (a zinc permease), while the efflux of zinc is accomplished by ZntA (a P-type ATPase) and ZitB (a cation diffusion facilitator) [7,8,12-21]. Within the cytoplasm, similar to copper, it is thought that zinc trafficking may involve chaperone-like proteins [22,23]. However, despite considerable experimental effort, the zinc chaperone protein in *E. coli *has yet to be identified [2,6,24-27]. The ZnuABC transporter (encoded by the *znuACB *gene cluster) is composed of the periplasmic binding protein ZnuA, the ATPase ZnuC, and the integral membrane protein ZnuB [28]. This zinc uptake system is regulated by Zur, a dimer protein which binds at least 2 zinc ions. Zur is sensitive to the intracellular zinc concentration, and zinc-bound Zur (presumably the Zn_4_Zur form, the Zur dimer which contains 2 zinc ions per monomer and it is denoted as Zn_2_Zur in [22]) can compete with RNA polymerase to bind to the *znu *operator and act as a repressor [7,8,20].

In contrast to this mechanism, zinc efflux through ZntA is regulated by ZntR, a zinc-responsive MerR-like transcriptional regulator [8,14,29,30]. ZntR is a dimer protein which can bind one or two zinc ions per monomer depending on the buffer conditions [29]. A metal occupancy assay of ZntR, monitored by changes in tyrosine fluorescence, shows non-cooperative 1:1 binding of Zn(II) to the ZntR dimer [31]. ZntR in its apo form only slightly activates *zntA *transcription (please note that the apo form of ZntR (i.e., apo-ZntR) means that ZntR without the binding of Zn(II)) [8,14,29]. The binding of zinc-bound ZntR to the promoter introduces conformational changes in the DNA, which apparently make the promoter a better substrate for RNA polymerase, thus strongly activating the transcription of the *zntA *gene and increasing the efflux of zinc from the cell [29].

**Figure 2:**
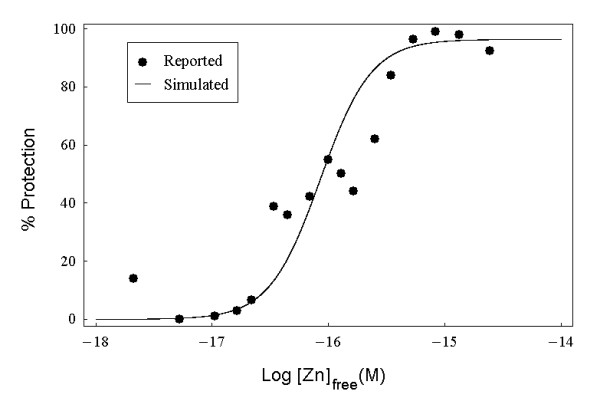
**Simulation of Zur-DNA interaction**. The black dots are reconstructed from the reported data in the original figure (the right graph in Fig. 3 in [22]) using image analysis method (please refer to the **Methods **for more details). The black curve is the simulated ratio (i.e., $Qw_{2}^{s}$/*D*_0 _* 100%) of the final steady state concentration values of Zn_4_Zur-DNA complex (denoted by $Qw_{2}^{s}$) and the total concentration of DNA (*D*_0 _= 1 *nM *in this case) as a function of the logarithm of parameter *Zn *which denotes the simulated free zinc concentration.

During 1999–2001, Outten CE and her colleagues presented some results on *in vitro *transcription and metal-binding competition experiments of *E. coli *zinc homeostasis system and showed that both ZntR and Zur are extremely avid zinc sensors and are both saturated at femtomolar free zinc concentrations [22,29,31]. In these experiments, the Zn(II) concentration was precisely controlled by using *N*,*N*,*N*',*N*'-tetrakis(2-pyridylmethyl) ethylenediamine (TPEN) as a zinc buffer [22]. The various assays relevant to this paper include the Zur-DNA interaction assay, Zur transcription assay and two ZntR transcription assays. In the Zur-DNA interaction assay the DNase I footprinting technique was used and the Zur-DNA interaction was found to correlate with the concentration of free Zn(II) (see the black dots in Fig. 2).

**Figure 3:**
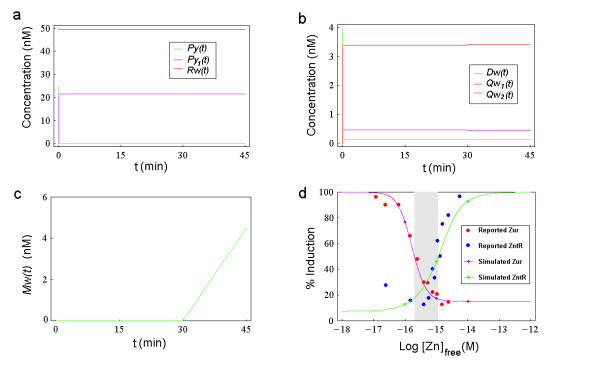
**Transient curves of simulated Zur transcription assay and data comparison (I)**. (a) The green, purple and red curves denote the simulated transient curves of Zn_2_Zur (*Py*), Zn_4_Zur (*Py*_1_), RNA polymerase (*Rw*) concentrations as a function of *t*, respectively. (b) The green, purple and red curves denote the simulated transient curves of free *znuC *DNA (*Dw*), *znuC *transcription initiation complex (*Qw*_1_) and Zn_4_Zur-DNA complex (*Qw*_2_) concentrations as a function of *t*, respectively. (c) The simulated concentration of the mRNA of ZnuC (*Mw*(*t*)) is depicted as a function of *t*. (d) Data comparison for Zur and ZntR transcription assays. Big red dots for the Zur transcription assay and big blue dots for the ZntR transcription assay (I) are reconstructed from the reported data in the original figure (Fig. 4 in [22]) using image analysis method. The purple curve and the green curve are the corresponding simulated normalized final concentrations of the mRNA of ZnuC (*Mw *(*t *= *t*_*d*0 _+ *t*_*d*_)) and the mRNA of ZntA (*Mz *(*t *= *t*_*d*0 _+ *t*_*d*_)) as a function of the logarithm of *Zn *(also denoted as [*Zn*]_*free*_), respectively. The three small purple dots on the purple curve are simulated data points for *Zn *= 10^-5 ^*nM*, 10^-6 ^*nM*, 10^-7 ^*nM*, respectively. The three small green dots on the green curve are simulated data points for *Zn *= 10^-5 ^*nM*, 10^-6 ^*nM*, 10^-7 ^*nM*, respectively (please note that the simulated transient curves of Zur and ZntR transcription assays for *Zn *= 10^-5 ^*nM *are shown in Fig. 3a-c and Fig. 4, respectively. More simulated transient curves for *Zn *= 10^-6 ^*nM*, 10^-7 ^*nM *are shown in Additional file 2: MoreTransientCurves.doc). The area highlighted in gray is the range of *Zn *between the half maximal induction points on the two simulated curves.

In the Zur transcription assay, *in vitro *run-off transcription experiments with Zur and the *znu *Zn(II) uptake system were conducted, and the levels of the *znuC *RNA transcript were reported to correlate with the free Zn(II) concentration (see the red dots in Fig. 3d) [22]. In these real run-off transcription experiments, various reactants (including *znuC *DNA template, Zur, Zn(II), RNAP and heparin, etc.) were added sequentially and allowed to equilibrate first (~30 min total). Then nucleoside triphosphates (NTPs) were added and the reaction was stopped for 15 min (Outten CE, personal communication). Similar run-off transcription experiments (the ZntR transcription assay (I)) were conducted with ZntR and the *zntA *promoter and the levels of the *zntA *RNA transcript were reported to correlate with the free Zn(II) concentration (see the blue dots in Fig. 3d) [22]. Similarly in ZntR transcription assay (II), the levels of the *zntA *RNA transcript were reported to correlate with the total ZntR concentration, both with added Zn(II) and without Zn(II) (see the red and blue dots in Fig. 5a, respectively). Moreover, it was also reported that the levels of the *zntA *RNA transcript correlated with the total zinc concentration (see the black dots in Fig. 5b) [29].

Although the transcriptional regulation of the zinc homeostasis system in *E. coli *seems to be well characterized, and despite the fact that detailed *in vitro *experimental data on this system are also available, as yet there is no mathematical model to help interpret these data [22,29,31]. The principal aim of this paper is to present a mathematical model which is capable of simulating this regulatory system and can be used to help interpret various experimental data.

We will present a unified mathematical model and use it to simulate the *in vitro *transcriptional response of the zinc homeostasis system in *E. coli*. The construction of the model is based on biochemical principles and we use open source software (Cellerator) to automatically generate the equations [32,33]. We validate our model by comparing the simulation results with the corresponding *in vitro *experimental data.

## Results

As shown in Table 1, we use 14 reactions to represent the four sub-processes involved in the zinc homeostasis system, namely: (i) Zn^2+^-sensing by Zur, (ii) transcriptional repression of the *znuC *gene by Zur, (iii) Zn^2+^-sensing by ZntR and (iv) transcriptional activation of the *zntA *gene by ZntR (see Fig. 1b, and for more details please refer to the **Methods**). Here we present our results for simulating various *in vitro *assays (please note that the main differences between the ZntR transcription assay (I) and assay (II) are differences in the initial conditions and in that in assay (II) we take into consideration competition between ZntR and TPEN for zinc binding by including Reaction 14, whereas in assay (I), Reaction 14 is not included).

**Table 1:** The reactions of the model

**Sub-Process **Name	**Reaction **No.	**Cellerator Form **of Particular Reactions	**Description**
Zn^2+^-Sensing by ZntR	(1)	{*Px *+ *Zn *⇄* Px*_1_, *r*_1_, *r*_2_}	apo-ZntR binding with zinc to become active ZntR

Transcriptional Activation of *zntA *Gene by	(2)	{*Dz *+ *Rz *⇄* Qz*_1_, *k*_2*a*_, *k*_-2_}	DNA of ZntA binding with RNAP
	(3)	{*Qz*_1 _⇄* Dz *+ *Mz *+ *Rz*, *k*_3_, 0}	transcription of *Qz*_1_
	(4)	{*Dz *+ *Px *⇄* Qz*_4_, *k*_1*b*_, *k*_-1_}	apo-ZntR binding with DNA
	(5)	{*Qz*_4_+ *Rz ⇄ Qz*_5_, *k*_2*b*_, *k*_-2_}	apo-ZntR-DNA complex binding with RNAP
	(6)	{*Qz*_5 _*⇄ Qz*_4_+ *Mz *+ *Rz*, *k*_3_, 0}	transcription of *Qz*_5_
	(7)	{*Dz *+ *Px*_1 _*⇄ Qz*_2_, *k*_1_, *k*_-1_}	ZnZntR binding with DNA
	(8)	{*Qz*_2 _+ *Rz ⇄ Qz*_3_, *k*_2*c*_, *k*_-2_}	ZnZntR-DNA complex Binding with RNAP
	**(9)**	{*Qz*_3 _⇄* Qz*_2_+ *Mz *+ *Rz*, *k*_3_, 0}	**transcription of ***Qz*_3_

Zn^2+^-Sensing by Zur	(10)	{*Zn*^2^+ *Py *⇄* Py*_1_, *r*_3_, *r*_4_}	Zn_2_Zur binding with zinc to become active Zur

Transcriptional Repression of *znuC *Gene by Zur	(11)	{*Dw *+ *Py*_1 _⇄* Qw*_2_, *k*_1*a*_, *k*_-1_}	active Zur binding with DNA to form complex *Qw*_2 _which can not bind with RNAP
	(12)	{*Dw *+ *Rw *⇄* Qw*_1_, *k*_2_, *k*_-2_}	DNA of ZnuC binding with RNAP
	(13)	{*Qw*_1 _⇄* Dw *+ *Mw *+ *Rw*, *k*_3_, 0}	transcription of *Qw*_1_

**Zn^2+^-Binding by TPEN**	**(14)**	{*Zn *+ *Tp *⇄* Tp*_1_, *r*_5_, *r*_6_}	**TPEN binding with zinc to form a complex**

Note: Abbreviations and synonyms used in this table are as follows: *Zn *(free zinc ion); *Px *(apo-ZntR); *Px*_1 _(active ZntR, i.e., ZnZntR); *Py *(the Zur dimer which contains two zinc ions per dimer, here we denote it as Zn_2_Zur and it is denoted as Zn_1_Zur in [22]); *Py*_1 _(active Zur, i.e., the Zur dimer which contains four zinc ions per dimer, here we denote it as Zn_4_Zur and it is denoted as Zn_2_Zur in [22]); *Z *(ZntA); *DZ *(DNA of ZntA); *Rz *(RNA polymerase for *zntA *transcription); *Mz *(mRNA of ZntA); *Qz*_1 _(transcription initiation complex formed by *Dz*and *Rz*); *Qz*_2 _(ZnZntR-DNA complex); *Qz*_3 _(transcription initiation complex formed by *Qz*_2 _and *Rz*); *Qz*_4 _(apo-ZntR-DNA complex); *Qz*_5 _(transcription initiation complex formed by *Qz*_4 _and *Rz*); *w *(ZnuC);*Dw *(DNA of ZnuC); *Rw *(RNA polymerase for *znuC *transcription); *Mw *(mRNA of ZnuC); *Qw*_1 _(transcription initiation complex of ZnuC); *Qw*_2 _(Zn_4_Zur-DNA complex which can not further bind with *Rw*); *Tp *(free TPEN not bound by zinc); *Tp*_1 _(zinc-bound TPEN); RNAP (RNA polymerase).

### Zur-DNA interaction

The Zur-DNA interaction assay involves only two reactions (Reactions 10 and 11, see Table 1), which are expressed as 4 ODEs (for the detailed equations, see Additional file 1: ModelEquations.doc) [22]. By imposing the conservation restraints (*Py*(*t*) + *Py*_1_(*t*) + *Qw*_2_(*t*) = *Py*_*tot *_= 25 *nM*, *Dw*(*t*) + *Qw*_2_(*t*) = *D*_0 _= 1 *nM*) (as in the real experiment [22]) and solving the relevant steady state equations (for the parameters, please see Table 2. Note that in the real experiment the total concentration of Zur monomer is 50 nM, here we need to divide this value by half which means that *Py*_*tot *_= 25 *nM *because in solution, Zur exists in dimer form [20]. In similar way we can calculate *Px*_*tot*_), we can depict the simulated ratio of steady state concentrations of the Zn_4_Zur-DNA complex (denoted by $Qw_{2}^{s}$) and the total concentration of *znuC *DNA (*D*_0 _= 1 *nM *in this case) as a function of the logarithm of parameter *Zn *as shown in Fig. 2 (the black curve). From this figure, we can see that when the simulated free zinc concentration (*Zn*) ranges from 10^-18 ^M to 10^-14 ^M, the simulated protection ratio (denoted by $Qw_{2}^{s}$/*D*_0 _* 100%) rises from 0.00014% to 96.4%. This means that in the presence of higher free zinc concentrations, more Zn_2_Zur molecules become active and bind with *znuC *DNA molecules to protect them from the binding of RNA polymerase. The simulated sigmoidal curve (the black curve in Fig. 2) seems to fit well with the corresponding experimental data (the black dots in Fig. 2) [22].

**Table 2:** Model parameters for which all results are calculated unless otherwise stated

**Parameter**	**Value**	**Description**
*k*_*d*_	10^-14.9 ^M	the Zn(II) dissociation constant for ZnZntR when pH = 8.0 [31]
*k*_*d*1_	10^-15.2 ^M	the Zn(II) dissociation constant for the ZnZntR-DNA complex when pH = 8.0 [31]
$K_{Zn-TPEN}^{'}$	1.99*10^15 ^M^-1^	the apparent association constant for Zn-TPEN at pH = 8.0, 0.1 M ionic strength, calculated from [22]
*k*_1_	0.025 (nM)^-1^s^-1^	the forward rate parameter of Reaction (7)
*k*_1*a*_	1 (nM)^-1^s^-1^	the forward rate parameter of Reaction (11)
*k*_1*b*_	1.253*10^-2 ^(nM)^-1^s^-1^	the forward rate parameter of Reaction (4)
*k*_-1_	0.9 s^-1^	the backward rate parameter of Reactions (4,7,11)
*k*_2_	0.02 (nM)^-1^s^-1^	the forward rate parameter of Reaction (12)
*k*_2*a*_	0.00005 (nM)^-1^s^-1^	the forward rate parameter of Reaction (2)
*k*_2*b*_	0.0002 (nM)^-1^s^-1^	the forward rate parameter of Reaction (5)
*k*_2*c*_	0.0037 (nM)^-1^s^-1^	the forward rate parameter of Reaction (8)
*k*_-2_	0.3 s^-1^	the backward rate constant of Reactions (2, 5, 8, 12)
*k*_3_	0.011 s^-1^	the transcription rate parameter
*r*_1_	2.73*10^2 ^(nM)^-1^s^-1^	the forward rate parameter of Reaction (1)
*r*_2_	3.437*10^-4 ^s^-1^	the backward rate parameter of Reaction (1)
*r*_3_	4.41*10^10 ^(nM)^-2^s^-1^	the forward rate parameter of Reaction (10)
*r*_4_	9*10^-3 ^s^-1^	the backward rate parameter of Reaction (10)
*r*_5_	3*10^4 ^(nM)^-1^s^-1^	the forward rate parameter of Reaction (14)
*r*_6_	1.506 *10^-2 ^s^-1^	the backward rate parameter of Reaction (14)
*t*_*d*0_	30 min	the time duration for preliminary equilibrium of reactants before NTPs (i.e., nucleoside triphosphates)were added in run-off transcription experiments [22,29,37]
*t*_*d*_	15 min	the time duration for run-off transcription after NTPs were added in transcription experiments [22,29,37]
*t*_*d*1_	30 min	the time duration for Zur-DNA interaction assay [22]
*Px*_*tot*_	25 nM	the total concentration of ZntR dimer which is half of the concentration of ZntR monomer denoted as [*ZntR*]_*total*_[22]
*Py*_*tot*_	25 nM	the total concentration of Zur dimer [22]
*R*_0_	50 nM	the total concentration of RNAP [22]
*D*_0_	4 nM	the total concentration of DNA [22]
*Zn*_*tot*_	vary in different assays	the total concentration of Zn(II), also denoted as [*Zn*]_*total*_
*TPEN*_*tot*_	vary in different assays	the total concentration of TPEN

Note: *k*_2*b *_= 4*k_2*a*_. Moreover, according to the equilibrium theory of chemical reactions, *r*_2 _= *k*_*d*_*r*_1_, *r*_6 _= *r*_5_/$K_{Zn-TPEN}^{'}$ and *k*_1*b *_= *k*_1_*k*_*d*_/*k*_*d*1_. The values of four parameters (*k*_1_, *k*_-1_, *k*_2_, *k*_-2_) are taken from Hayot's model [43]. These parameters origin from measured rate constants of the *λ *repressor gene cI in *E. coli *and are also quoted as physiologically reasonable values by Ingram *et al *[15,43,44]. $K_{Zn-TPEN}^{'}$ is calculated in the same way as shown in [22] (please note the pH value difference).

We derive the same simulation results by directly solving the 4 relevant ODEs with *Py*(0) = *Py*_*tot *_= 25 *nM*, *Dw*(0) = *D*_0 _= 1 *nM*, *Dw*_2_(0) = 0, *Py*_1 _(0) = 0 as the initial conditions and depicting the simulated ratio of the final concentration of the Zn_4_Zur-DNA complex (*Qw*_2_(*t *= *t*_*d*1_) and *D*_0_. This is because the system reaches equilibrium before *t *= *t*_*d*1 _= 30 min.

### Zur transcription assay

As mentioned in the legend of Fig. 1b, here we only simulate the transcription of the *znuC *gene. We approximate the *in vitro *Zur run-off transcription assay by a two-phase (namely, the preliminary equilibrium phase and the transcription phase) sub-model. In the first phase, the preliminary equilibrating process of reactants involves 3 reactions (Reactions 10–12) which are expressed as 6 ODEs (see Additional file 1: ModelEquations.doc). In the second phase, the run-off transcription involves 4 reactions (Reactions 10–13 because now the real transcription happens after the addition of the NTPs) which are expressed as 7 ODEs (see Additional file 1: ModelEquations.doc).

By setting the initial conditions of the model simulation to be the same as those in the real experiment (*Py*(0) = *Py*_*tot *_= 25* nM*, *Dw*(0) = *D*_0 _= 4 *nM*, *Rw*(0) = *R*_0 _= 50 *nM *and all the remaining initial concentrations are set to be 0) and numerically solving the 6 equations for the first phase and then solving the 7 ODEs for the second phase (obviously we need to use the end concentration values of the reactants in the first phase as the initial concentration values of reactants in the second phase), we can depict the relevant transient curves for *Zn *= 10^-5 ^as shown in Fig. 3a–c (for the values of the remaining parameters, please see Table 2).

As shown in Fig. 3a, due to the binding of free zinc, the simulated concentration of Zn_2_Zur (*Py*(*t*), the Zur dimer which contains two zinc ions per dimer and it is used in the corresponding real assay [22]) quickly decreases from 25 nM to a steady state value of 0.044 nM whereas the simulated concentration of active Zur (*Py*_1 _(*t*), the Zur dimer which contains four zinc ions per dimer) quickly rises from 0 to 21.6 nM. The simulated concentration of RNA polymerase (*Rw*(*t*)) decreases slightly from 50 nM to 49.5 nM due to the effect of its binding with *znuC *DNA.

As we can see from Fig. 3b, the simulated free *znuC *DNA concentration (*Dw*(*t*)) decreases rapidly (in 0.4 seconds) from 4 nM to a steady state of 0.14 nM during the first phase due to the binding of active Zur and RNA polymerase. The simulated concentration of the transcription initiation complex (*Qw*_1_(*t*)) rapidly increases (in 0.4 seconds) from 0 to a steady state value of 0.47 nM whereas the simulated concentration of Zn_4_Zur-DNA complex (*Qw*_2_(*t*)) quickly increases (in 0.6 seconds) from 0 to 3.39 nM. The initiation of the second phase seems to only have a small influence on the afore mentioned steady state values (e.g., the steady state values of *Qw*_1_(*t*) and *Qw*_2_(*t*) change from 0.47 nM and 3.39 nM at the end of first phase to 0.45 nM and 3.4 nM at the end of the second phase, respectively). From Fig. 3c, we can see that in the first 30 minutes, the concentration of mRNA of ZnuC (*Mw*(*t*)) remains at 0 because the real transcription has not happened yet, and then in the subsequent 15 minutes it increases linearly from 0 to a final concentration of 4.49 nM.

The rapid decrease in the concentration of free *znuC *DNA (*Dw*(*t*)) shown in Fig. 3b is due to the binding of *znuC *DNA with active Zur (Zn_4_Zur) and RNA polymerase. Since in the whole process, the total increase in the simulated concentration of Zn_4_Zur-DNA complex (*Qw*_2_(*t*)) is 3.4 nM, whereas the total decrease of the simulated free *znuC *DNA concentration is about 3.86 nM, we can conclude that when *Zn *= 10^-5 ^*nM*, the binding of active Zur consumes the majority of the *znuC *DNA to form the Zn_4_Zur-DNA complex, which can not further bind with RNA polymerase, and in this way the transcription of *znuC *is repressed.

We performed many simulations for various values of *Zn *(in the range of 10^-18 ^M to 10^-12 ^M) and recorded the final values of the simulated mRNA concentration (*Mw*(*t *= *t*_*d*0 _+ *t*_*d*_)). After normalizing these concentration values, depicting them as a function of *Zn *(in logarithm), and smoothly connecting these simulated data points, we obtained the purple curve in Fig. 3d (please note that only three simulated data points for Zur assay are shown as small purple dots in this figure to avoid confusion with the experimental data points).

### ZntR transcription assay (I)

**Figure 4:**
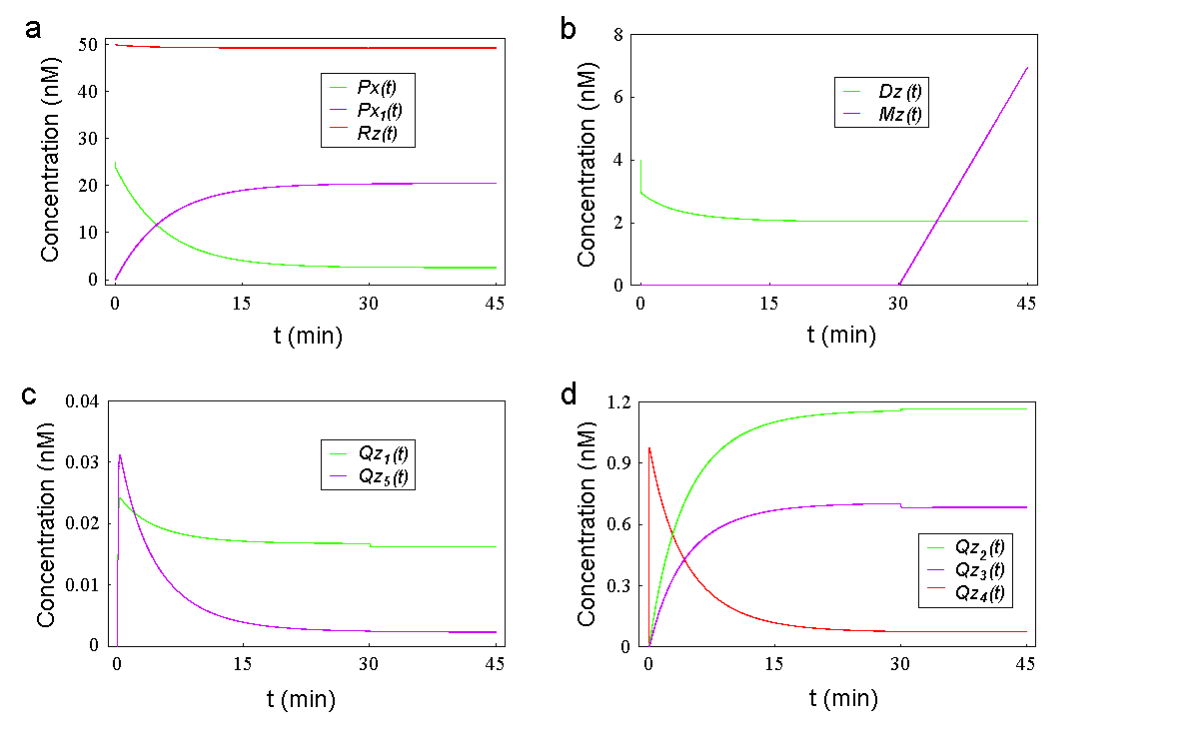
**Transient curves of simulated ZntR transcription assay (I)**. (a) The green, purple and red curves denote the simulated transient curves of apo-ZntR (*Px*), ZnZntR (*Px*_1_), RNA polymerase (*Rz*) concentrations as a function of *t*, respectively. (b) The simulated concentrations of the free *zntA *DNA (*Dz*, green curve) and mRNA of ZntA (*Mz*, purple curve) are depicted as a function of *t*. (c) The green and purple curves denote the simulated transient curves of transcription initiation complexes (*Qz*_1 _and *Qz*_5_) concentrations as a function of *t*, respectively. (d) The green, purple and red curves denote the simulated transient curves of ZnZntR-DNA complex (*Qz*_2_), transcription initiation complex (*Qz*_3_) and apo-ZntR-DNA complex (*Qz*_4_) as a function of *t*, respectively.

Similarly, the ZntR run-off transcription assay can also be simulated by a two-phase sub-model. The first phase (the preliminary equilibrium phase) involves 6 reactions (Reactions 1,2,4,5,7,8), which are expressed as 9 ODEs (see Additional file 1: ModelEquations.doc). The second phase (the transcription phase) involves 9 reactions (Reactions 1–9), which are expressed as 10 ODEs (see Additional file 1: ModelEquations.doc). By setting the initial conditions of the simulation to be the same as those used in the real experiment (*Px*(0) = *Px*_*tot *_= 25 *nM*, *Dz*(0) = *D*_0 _=4 *nM*, *Rz*(0) = *R*_0 _= 50 *nM *and setting all the remaining initial concentrations to be 0), and subsequently solving the relevant equations of the two-phase sub-model, we can depict the relevant transient curves for *Zn *= 10^-5 ^*nM *as shown in Fig. 4 (for the remaining parameters, please see Table 2). In this assay, Reaction 14 is not included in the sub-model because the ZntR concentration is too low to challenge the buffering capacity of TPEN (of course we can also perform numerical simulations by including Reaction 14, although further investigations have shown that we essentially get the same results).

From Fig. 4a we can see that due to the binding with free zinc, the simulated concentration of apo-ZntR (*Px*(*t*)) decreases from 25 nM to 2.59 nM, whereas the simulated concentration of active ZntR (*Px*_1_(*t*)) rises from 0 to 20.5 nM, and the simulated concentration of RNA polymerase (*Rz*(*t*)) decreases slightly from 50 nM to 49.3 nM. In the first phase, due to the binding with ZntR and RNA polymerase, the simulated unbound *zntA *DNA concentration (*Dz*(*t*)) decreases rapidly (in 0.04 minutes) from 4 nM to 3.03 nM and then decreases gradually to 2.04 nM at the end of the first phase (Fig. 4b, green curve); in the second phase, the free *zntA *DNA concentration remains at roughly the same level (2.05 nM). The simulated *zntA *mRNA concentration (*Mz*(*t*)) remains at 0 nM during the first phase, as there is no transcription happening, and then increases seemingly linearly to a final concentration of 6.96 nM during the second phase after NTPs have been added (Fig. 4b, purple curve).

The simulated transients curves in Fig. 4c show that *Qz*_1_(*t*) rapidly rises (in 0.3 minutes) from 0 to a peak value of 0.024 nM and then gradually decreases to 0.017 nM during the first 30 minutes whereas *Qz*_5_(*t*) rapidly rises (in 0.26 minutes) from 0 to a peak value of 0.031 nM and then gradually decreases to a 0.0025 nM during the first phase. The initiation of the second phase causes a small decrease in the values of *Qz*_1_(*t*) and *Qz*_5_(*t*) (to 0.016 nM and to 0.0023 nM, respectively). As shown in Fig. 4d, both *Qz*_2_(*t*) and *Qz*_3_(*t*) rise first (from 0 to 1.16 nM and 0.7 nM, respectively) during the first phase whereas *Qz*_4_(*t*) first dramatically increases up to a peak value of 0.97 nM and then gradually decreases to its final value of 0.076 nM. The initiation of the second phase causes a small decrease in the value of *Qz*_3_(*t*) and a slight increase in the value of *Qz*_2_(*t*), as judged by the small kinks in the corresponding two curves, whereas it has insignificant influence of the value of *Qz*_4_(*t*).

Using similar methods we can obtain the green curve in Fig. 3d for the final values of the simulated *zntA *mRNA concentration (*Mz*(*t *= *t*_*d*0 _+ *t*_*d*_)) as a function of the value of *Zn *(in logarithm). The results shown in Fig. 3d indicate that when the simulated free zinc concentration ranges from 10^-18 ^M to 10^-12 ^M, the simulated normalized final concentrations of mRNA of ZnuC (*Mw*(*t *= *t*_*d*0 _+ *t*_*d*_)) decreases from 100% to 15.05%, whereas the simulated normalized final concentration of mRNA of ZntA (*Mz*(*t *= *t*_*d*0 _+ *t*_*d*_)) increases from 7.4% to 100%. The half-maximal induction of *znuC *transcripts and the half maximal induction of *zntA *transcripts occur at *Zn *= 2*10^-16 ^*M *and *Zn *= 1.15*10^-15 ^*M *respectively, which are the same as previously reported values [22]. The simulated purple curve (for the Zur transcription assay) agrees with corresponding experimental data (the red dots) extremely well. Similarly the simulated green curve (for the ZntR transcription assay (I)) also agrees with the corresponding experimental data (the blue dots), although to a slightly lesser degree [22].

### ZntR transcription assay (II)

**Figure 5:**
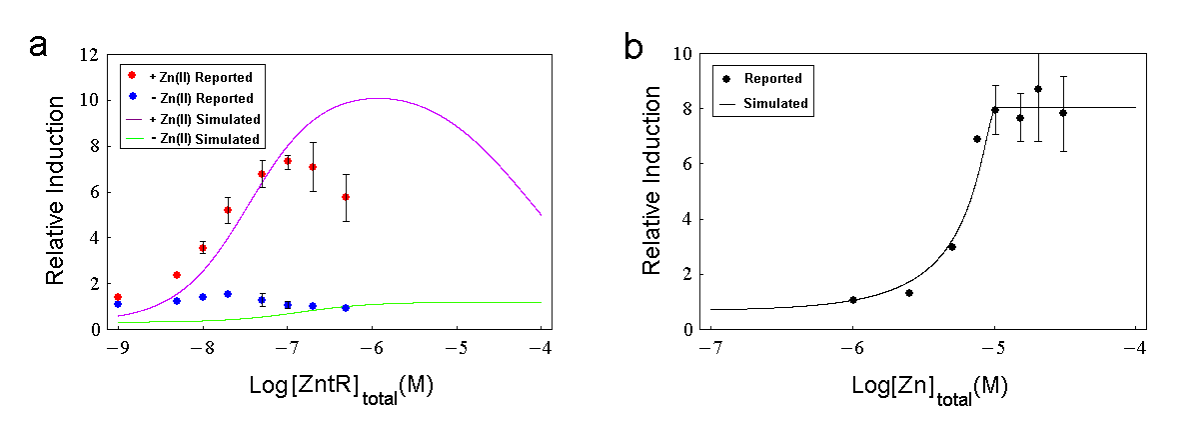
**Comparison of simulated results and experimental data (II)**. (a) ZntR transcription assay with Zn(II) or without Zn(II). Red dots for the case of with Zn(II) and green dots for the case of without Zn(II) are reconstructed from the reported data in the original figure (Fig. 6B in [29]) using image analysis. *Error bars *indicate a standard deviation both above and below the average values of two separate experiments. The purple line and the green line are the corresponding simulated final concentrations (in nM) of mRNA of ZntA (*Mz*(*t *= *t*_*d*0 _+ *t*_*d*_)) in the cases of parameter *Zn*_*tot *_= 10 μ*M *and *Zn*_*tot *_= 0 as a function of the logarithm of [*ZntR*]_*total *_(i.e., 2*Px_*tot*_), respectively. (b) ZntR transcription assay with varying total zinc concentration. The black dots are reconstructed from the reported data in the original figure (Fig. 6C in [29]) using image analysis. The black curve is the simulated final concentration (in nM) of mRNA of ZntA (*Mz*(*t *= *t*_*d*0 _+ *t*_*d*_)) as a function of the logarithm of parameter *Zn*_*tot *_(also denoted as [*Zn*]_*total*_).

In this assay, we take into consideration the competition between ZntR and TPEN for zinc binding by including Reaction 14. Again, we will use a two-phase sub-model to simulate the real assay. The first phase (the preliminary equilibrium phase) of the assay (II) involves 7 reactions (Reactions 1,2,4,5,7,8,14), which are expressed as 12 ODEs (see Additional file 1: ModelEquations.doc). The second phase (the transcription phase) involves 10 reactions (Reactions 1–9,14), which are expressed as 13 ODEs (see Additional file 1: ModelEquations.doc). By setting the initial conditions of the simulation equal to those used in the real experiment ($\begin{array}{l} Tp(0)=TPEN_{tot}=10\mu M,Zn(0)=Zn_{tot},Px(0)=Px_{tot},\\ Dz(0)=D_{0}=2nM,Rz(0)=R_{0}=100nM \end{array}$, and all the remaining initial concentrations are set to 0) and solving the two-phase model, we depict the simulated final concentrations (in nM) of mRNA of ZntA (*Mz*(*t *= *t*_*d*0 _+ *t*_*d*_)) for *Zn*_*tot *_= 10 μ*M *and *Zn*_*tot *_= 0 as a function of the logarithm of the doubled value of parameter *Px*_*tot *_(i.e., [*ZntR*]_*total *_which denotes the total concentration of the ZntR monomer) and we obtain the purple and green curves shown in Fig. 5a[29].

We also perform many simulations under the following initial conditions: ($\begin{array}{l} Tp(0)=TPEN_{tot}=10\mu M,Zn(0)=Zn_{tot},Px(0)=Px_{tot}=50nM,\\ Dz(0)=D_{0}=2nM,Rz(0)=R_{0}=100nM \end{array}$, and all the remaining initial concentrations are set to 0) for various values of *Zn*_*tot *_within the range of 100 nM to 100 μM and eventually obtain the black curve shown in Fig. 5b which describes the final values of the simulated mRNA concentration (*Mz*(*t *= *t*_*d*0 _+ *t*_*d*_)) as a function of the value of *Zn*_*tot *_(in logarithm).

## Discussion

The simulation results shown in Fig. 4 indicate the complex interactions among three transcription processes of *zntA *(the constitutive transcription, the apo-ZntR activated transcription and the ZnZntR activated transcription). If we compare the dynamics of the simulated concentrations of three transcription initiation complexes involved in the ZntR transcription assay (i.e., *Qz*_1_(*t*), *Qz*_3_(*t*) and *Qz*_5_(*t*)) as shown in Fig. 4c and Fig. 4d, we find that the dynamics of *Qz*_1_(*t*) and *Qz*_5_(*t*) are quite similar. Initially, they both increase rapidly, form low peaks (the peak values are 0.024 nM and 0.031 nM, respectively), and then gradually decrease. In contrast, the dynamics of *Qz*_3_(*t*) only demonstrates a gradual increase to 0.7 nM in the first 30 minutes. The observation that the final steady state value of *Qz*_3_(*t*) (0.69 nM) is much higher than those of *Qz*_1_(*t*) and *Qz*_5_(*t*) (0.016 nM and 0.0023 nM, respectively) indicates that for *Zn *= 10^-5^*nM*, when the system (excluding *Mz*(*t*)) enters its final equilibrium, the dominating transcription process is ZnZntR activated transcription rather than the other two transcription processes (i.e., the constitutive transcription and the apo-ZntR activated transcription, please refer to **Methods **for more details).

To explain why the dynamics of *Qz*_1_(*t*) shows a peak, we suggest that the initial increase of *Qz*_1_(*t*) is due to the binding of *zntA *DNA with RNA polymerase. Then following the conversion of apo-ZntR to active ZntR by zinc-binding (see the green and purple curves in Fig. 4a), active ZntR binds with *zntA *DNA to form the ZnZntR-DNA complex (see the green curve in Fig. 4b and the green curve in Fig. 4d). This competitive binding of active ZntR causes a sudden decrease in the free *zntA *DNA concentration (see the green curve in Fig. 4b) and the reversible Reaction 2 (see Table 1) becomes dominated by its reverse side and *Qz*_1_(*t*) begins to decrease after forming a small peak. Similarly, we can explain the dynamics of *Qz*_5_(*t*).

By comparing the dynamics of the simulated Zur and ZntR transcription assays shown in Fig. 3a–c and Fig. 4, we can see that when *Zn *= 10^-5^*nM*, during the first phase, the simulated Zur transcription system reaches its steady state in less than 20 seconds, much faster than the simulated ZntR transcription system which takes more than 20 minutes. As shown in Fig. 3c and Fig. 4b, the seemingly linear increase of the simulated concentrations of mRNA (*Mw*(*t*) and *Mz*(*t*)) during the second phase indicates the progress of the relevant transcription processes. If we calculate the slope of the linear curve in Fig. 3c as follows:

*Mw*(*t *= *t*_*d*0 _+ *t*_*d*_)/$Qw_{1}^{s}$/*t*_*d *_= 4.49 *nM*/0.45 *nM*/15 min = 0.011*s*^-1 ^where $Qz_{1}^{s}$ denotes the final steady state value of *Qz*_1_(*t*), we derive the same value as that of the transcription rate parameter *k*_3_. Obviously the simulated final concentrations of mRNA (*Mw*(*t *= *t*_*d*0 _+ *t*_*d*_) and *Mz*(*t *= *t*_*d*0 _+ *t*_*d*_)) are generally proportional to *t*_*d*_, which is in accordance with the experimental observation that the harvest of run-off transcription assay is related to the duration time of its transcription phase (*t*_*d*_) [34].

The purple curve in Fig. 5a indicates that for *Zn*_*tot *_= 10 μ*M*, when the simulated total ZntR monomer concentration ([*ZntR*]_*total *_which is twice the value of *Px*_*tot*_) ranges from 10^-9 ^M to 10^-4 ^M, the simulated final concentrations of mRNA of ZntA (*Mz*(*t *= *t*_*d*0 _+ *t*_*d*_)) increases from 0.59 nM to a peak value of 10.09 nM when [*ZntR*]_*total *_= 10^-5.92^*M *and then decreases to 4.99 nM. If we look at the corresponding experimental data (the red dots), we can see that the relative induction of the *zntA *transcripts increases, forms a peak (when [*ZntR*]_*total *_= 10^-7^*M*), and eventually declines [29]. Thus our simulation successfully simulates the peak behaviour of the relative induction of the *zntA *transcripts for increasing values of [*ZntR*]_*total *_in the presence of zinc. Further investigations show that if we perform the simulations excluding Reaction 14, then we can only reproduce the increasing behaviour rather than the peak behaviour. Thus one potential explanation for the peak behaviour is that, for low ZntR concentrations, TPEN is strong enough to buffer the zinc and more ZntR will promote the transcription of *zntA *gene; while for high ZntR concentrations, the buffering capacity of TPEN is exceeded and the free zinc concentration can not be maintained as a constant anymore and it subsequently decreases due to the binding of over-abundant ZntR molecules, which in turn limits the transcription processes. A similar comparison can be made for the case when *Zn*_*tot *_= 0 (i.e., in the absence of zinc, please see the green curve and the blue dots in Fig. 5a). However, in the latter case, our model can only simulate the initial increase, but fails to reproduce the decline.

As described in detail in the **Methods **section, in this model, we assume that the active form of ZntR is ZnZntR because metal occupancy assays of ZntR monitored by changes in tyrosine fluorescence show noncooperative 1:1 binding of Zn(II) to the ZntR dimer [31]. This assumption is valid only when the free zinc concentration and total ZntR concentration are both extremely low (in sub-nM and nM range, respectively). When the total ZntR concentration goes to the μM range, the binding kinetics of Zn(II) to the ZntR dimer will be more complicated because ZntR can bind one or two zinc ions per dimer depending on the buffer conditions [8,29]. This explains why, as shown in Fig. 5a in the case of with Zn(II), there is a disagreement between the simulation results (the purple curve) and the corresponding experimental data (the red dots) when ZntR molecules are relatively abundant. Intuitively, we can think of it in this way: in the real case, the competitive ability of ZntR for Zn(II) binding is stronger than the model prediction because at high ZntR concentrations, ZntR, on average, binds with more than one ion per dimer. This results in a smaller and earlier peak because the buffering capacity of TPEN is now easier to exceed. In the absence of Zn(II), the eventual abnormal decline in the experimental data (see the blue dots in Fig. 5a) may be due to the normal deviations of the different experiments because the levels of *zntA *transcript are very low in this case or perhaps this is due to some novel, unknown mechanisms (please note that the error bars shown in Fig. 5a indicate the standard deviation from the average values of only two separate experiments and there are only two data points having error bars for the case of without Zn(II)) [29].

As we can see from Fig. 5b, when the simulated total zinc concentration (*Zn*_*tot*_) ranges from 10^-7 ^M to 10^-4 ^M, the simulated final concentration of mRNA of ZntA (*Mz*(*t *= *t*_*d*0 _+ *t*_*d*_)) increases from 0.72 nM to 8.04 nM (saturation occurs when *Zn*_*tot *_= 10^-5^*M*) which means that more abundant free zinc ions bind with ZntR to promote activation of the transcription of the *zntA *gene. The simulated curve (the black curve) fits the experimental data (the black dots) quite well [29].

Coupled feedback loops have been recently recognized as essential building blocks of cellular networks [35]. The zinc homeostasis system in *E. coli *is a good example of such a building block because it follows from Fig. 1a that Zur and ZnuC form a 'negative circuit', since active Zur represses *znuC *(negative action) while zinc influx via ZnuC leads to larger amounts of active Zur molecules (positive action). Similar considerations point towards the negative circuit wiring between ZntR and ZntA. It is believed that such coupled negative feedback loops are quite helpful for enhancing homeostasis [35].

As previously mentioned, cytoplasmic zinc trafficking in *E. coli *may involve chaperone-like proteins whose existence is still being debated [2,6,22]. Outten *et al. *demonstrated *in vitro *that ZntR and Zur are sensitive to very low concentrations (femtomolar) of free zinc (also see Fig. 3d), therefore they proposed that free zinc in the cytosol of *E. coli *is not physiologically available under normal growth conditions [22]. Our simulation results further confirm their experimental data and support their proposal. However, in order to better understand the *in vivo *transcriptional regulation mechanisms of zinc homeostasis, further investigations are required to simulate the *in vivo *transcription processes and their responses to various environmental conditions.

Up until now, performing well-designed *in vitro *experiments has been one of the common ways used to infer the various characteristics of the corresponding *in vivo *systems. The current work provides a good example of how to use a unified mathematical model to explain complicated datasets obtained from *in vitro *metal-binding and transcription experiments which have been widely performed for metal ion homeostasis and detoxification systems [22,29,31,36,37]. The repression of Zur on the transcription of *znuACB *gene cluster and the activation of ZntR on the *zntA *transcription constitute the critical parts of the regulatory mechanisms of the zinc homeostasis system in *E. coli *(see Fig. 1a). This means that if we want to make predictive and useful model for the *in vivo *zinc homeostasis system, we need to model these transcriptional regulations. Although the current model only simulates the *in vitro *kinetics, together with its fitted rate constants it can be used as a good basis and reference for the future modelling of the corresponding *in vivo *system. Moreover, the quantitative distinguishment of the three transcription processes of *zntA *(the constitutive transcription, the apo-ZntR activated transcription and the ZnZntR activated transcription) in our model will be quite meaningful for modelling the *in vivo *system and it provides the possibility of including any additional regulations on these three processes which do happen *in vivo *[14,29].

In order to further our understanding of the process of zinc homeostasis in *E. coli*, the most critical thing is to identify the intracellular zinc chaperone, which is very likely to exist. Recently, proteomics has progressed to such a stage that it can determine the cellular response to any perturbation at the level of protein activation [38-40]. Thus mass spectrometry-based proteomics can be used to search the possible molecular candidates in addition to genome-wide high-throughput screens [25]. Once the zinc chaperone has been identified, the next step will be to measure the interactions between the zinc chaperone and the membrane transport proteins (ZnuABC, ZupT, ZntA and ZitB) and the interactions between the zinc chaperone and the metalloregulatory proteins (ZntR and Zur). Since similar work has already been done for the copper homeostasis system in *E. hirae*, the same equipments and experimental techniques used there (e.g., surface plasmon resonance analysis) can also be used to measure the kinetics of these interactions in the zinc homeostasis system [23,41].

Furthermore, we need to further take into consideration zinc storage and zinc utilisation by proteins in *E. coli *and quantify the concentrations of the relevant proteins, DNAs and mRNAs. Finally, the subtle details of relevant regulatory processes (e.g., proteolysis which has been proven to play a role) need to be further investigated [8]. Once we have characterised these processes in detail and have made corresponding sub-models for them, we can then integrate these sub-models together with the current model, in order to build a comprehensive model to describe the entire *in vivo *system. Further experiments determining the *in vivo *cellular response to various perturbations will be necessary for checking the validity of the model and also for model refinement. In this way, step by step we will acquire a complete map of the zinc homeostasis system in *E. coli *and reach a full understanding of the system dynamics. Close cooperation between pioneering experimentalists and computational scientists through iterative systems biology procedure (model → experiments → model) will be necessary for achieving such ambitious goals [42].

## Conclusion

To summarize, we have built a mathematical model for simulating the *in vitro *transcriptional response of zinc homeostasis system in *E. coli*. Simulation results show that our model can quantitatively reproduce the various results of the *in vitro *experiments conducted by Outten CE and her colleagues. Our model gives a detailed insight into the involved system dynamics and provides a general framework for simulating *in vitro *metal-binding and transcription experiments and interpreting relevant experimental data.

## Methods

### Cellerator software

Cellerator™ is a Mathematica^® ^package designed to facilitate biological modeling via automated equation generation [32,33]. It uses an arrow-based reaction notation to represent biochemical networks and is especially amenable for simulating signal transduction networks. For example, a reversible biochemical reaction (*A *+ *B *⇌ *C*, which means reactant *A *binds with reactant *B *to form product *C*, can be represented as {*A *+ *B ⇄ C*, *r*_*f*_, *r*_*b*_} in Cellerator form where *r*_*f *_and *r*_*b*_denote the forward and the backward rate constants, respectively. The detailed ODE notation of this reaction is: $\frac{dC}{dt}=-\frac{dA}{dt}=-\frac{dB}{dt}=-r_{b}C+r_{f}AB$.

### Representation of relevant reactions

As we can see in Fig. 1b, in order to simulate the *in vitro *transcriptional response, we need to model the four sub-processes involved:

#### 1) Zn^2+^-sensing by ZntR

ZntR is a dimer protein which can bind one or two zinc ions per dimer depending on the buffer conditions [8,29]. However, metal occupancy assay of ZntR monitored by changes in tyrosine fluorescence shows non-cooperative 1:1 binding of Zn(II) to the ZntR dimer [31]. The zinc-bound form of ZntR has been reported to contain 0.75 ± 0.075 zinc/monomer, neither favoring 1:1 binding nor 1:2 binding [8]. However, this result was obtained under the condition of excessive ZntR protein (5 μM) [8]. Since the free zinc concentration and total ZntR concentration are both extremely low (in sub-nM and nM range, respectively) in all the relevant real assays (except the ZntR transcription assay (II) related to Fig. 5a) of this paper, here we assume that the active form of ZntR is ZnZntR (i.e. there is a 1:1 binding) [22,29]. We use Reaction (1) (see Table 1) to describe this sub-process.

#### 2) Transcriptional activation of zntA gene by ZntR

Experimental results have shown that there is constitutive transcription activity of the *zntA *promoter [14]. According to Hayot *et al.*, this constitutive transcription can be described by Reactions (2–3) (see Table 1, please note that the justification for the specific parameter values used in Hayot's model can be found in [15]. Hayot's model is later used by Ingram *et al. *to study the dynamics of the bi-fan motif) [43,44].

In the absence of Zn(II), apo-ZntR binds to the promoter and distorts the DNA which appears to result in an approximately fourfold induction [14]. According to Hayot *et al.*, this apo-ZntR activated transcription can be described by Reactions (4–6) (see Table 1) and we have the relation: *k*_2*b *_= 4* *k*_2*a *_[43].

The binding of Zn(II) to ZntR converts it into a transcriptional activator protein that introduces conformational changes in the DNA which apparently make the promoter a better substrate for RNA polymerase [29]. According to Hayot *et al.*, this ZnZntR activated transcription can be described by Reactions (7–9) (see Table 1) [43].

#### 3) Zn^2+^-sensing by Zur

Zur is a dimer protein which binds at least 2 zinc ions [20,22]. Experimental results have established that the DNA binding of Zur presumably involves the Zn_4_Zur form (i.e., the Zur dimer which contains 2 zinc ions per monomer and it is denoted as Zn_2_Zur in [22]) rather than the Zn_2_Zur form (the Zur dimer which contains one zinc ion per monomer and it is denoted as Zn_1_Zur in [22]). Similar as Cui *et al. *did for modelling the binding of calmodulin with calcium ions, we use Reaction (10) (see Table 1) to describe this sub-process under the assumption of strong cooperativity existing between the two active sites of Zn_2_Zur (please note that the purified Zur dimer which contains one zinc ion per monomer is used in the relevant assays) [22,45].

#### 4) Transcriptional repression of znuC gene by Zur

The genes *znuA *and *znuCB *are transcribed divergently and both promoters of *znuA *and *znuCB *are active *in vivo *[7,22]. Since we only have reported data for *znuC *transcripts available for comparison, here we choose to model the transcription of the *znuC *gene only [22]. In the absence of Zn(II), Zur does not compete for DNA binding. The addition of excessive Zn(II) allows Zur to bind to the *znuC *promoter and prevents its binding with RNA polymerase [22]. According to Hayot *et al.*, we can use Reactions (11–13) (see Table 1) to describe this process [43].

### Zinc binding by TPEN

As mentioned before, TPEN is used as a zinc buffer to precisely control the free zinc concentration in the relevant assays and this process can be apparently described by Reaction 14 [22,29]. Normally the free zinc concentration (*Zn*) is regarded as a constant and it can be simply calculated from the total zinc concentration (*Zn*_*tot*_) according to the following buffer equation:

$$Zn*(TPEN_{tot}-(Zn_{tot}-Zn))/(Zn_{tot}-Zn)=1/K_{Zn-TPEN}^{'}.$$

However, in more complicated cases such as the ZntR transcription assay (II), it is wiser to perform numerical simulations by including this reaction and the free zinc concentration is no longer regarded as a constant.

### The equations of the model and the numerical solver

The detailed equations used for simulating different assays can be found in Additional file 1: ModelEquations.doc. We use Mathematica's differential equation solver "NDSolve" to solve the relevant ODEs. If the studied ODEs are stiff as is the case for the relevant simulations of Fig. 5, we set the method option of NDSolve to be "StiffnessSwitching".

### Translating the model into CellML

CellML is an XML-based modelling language which provides an unambiguous method of defining models of biological processes [46,47]. The current model has been translated into two CellML versions [48,49]. The first version (please visit the webpage for downloading the detailed code) is for ZntR transcription assay (I) which excludes the buffering equation of TPEN (i.e., Reaction 14) [48]. The second version (please visit the webpage for downloading the detailed code) is for ZntR transcription assay (II) which includes the buffering reaction of TPEN [49].

### The image analysis method

The original figures are imported into the Paint tool of Windows system. The pixel coordinates are recorded for the axis origin, two tick points (one tick point on the horizontal axis and one tick point on the vertical axis) and all experimental data points. Then by simple algebraic calculations we can get the real coordinate values of the reported data points. For example, imagine that we need to analyze an image with x coordinate (in logarithm) and normal y coordinate. Assume the measured pixel coordinates of the axis origin (its real coordinate values are {10^a^, b}) and tick points (their real coordinate values are {10^a^, c} and {10^d^, b}) are (p_x0_, p_y0_), (p_x0_, p_y1_), (p_x1_, p_y0_), respectively. For a data point with measured pixel coordinates (p_x2_, p_y2_), we can calculate its real coordinate values {f, g} as follows:

$$f=10^{\left(d-a)*(p_{x2}-p_{x0})/(p_{x1}-p_{x0}\right)},g=(c-b)*(p_{y2}-p_{y0})/(p_{y1}-p_{y0})$$

The relative error of such data reconstruction is estimated to be (0.5–3)% depending on the image size.

## Authors' contributions

JC conceived of the study, participated in its design, performed numerical simulations and helped to draft the manuscript. JAK participated in its design and helped to draft the manuscript. CML translated the model into CellML versions and helped to draft the manuscript. All authors read and approved the final manuscript.

## Supplementary Material

Additional file 1 **ModelEquations.doc**. This additional file describes the detailed equations for simulating various assays. Click here for file

Additional file 2 **MoreTransientCurves.doc**. This additional file describes the simulated transient curves of Zur and ZntR transcription assays for parameter *Zn *= 10^-6^*nM*, 10^-7^*nM*. Click here for file
