# Supplementary material for: Simulating in vitro transcriptional response of zinc homeostasis system in Escherichia coli
Source: BMC Syst Biol. 2008 Oct 24;2:89. doi: 10.1186/1752-0509-2-89 (PMC2611976; doi:10.1186/1752-0509-2-89)
Supplement: Additional file 1 — ModelEquations.doc. This additional file describes the detailed equations for simulating various assays. [file 1752-0509-2-89-S1.doc]

## Abbreviations and synonyms used in this additional file are as follows: $Zn$(free zinc ion); $Px$(apo-ZntR); $Px_{1}$(active ZntR, i.e., ZnZntR); $Py$( the Zur dimer which contains two zinc ions per dimer, here we denote it as Zn_2_Zur and it is denoted as Zn_1_Zur in [26]); $Py_{1}$(active Zur, i.e., the Zur dimer which contains four zinc ions per dimer, here we denote it as Zn_4_Zur and it is denoted as Zn_2_Zur in [26]); $z$(ZntA); $Dz$(DNA of ZntA); $Rz$(RNA polymerase for *zntA* transcription); $Mz$(mRNA of ZntA); $Qz_{1}$(transcription initiation complex formed by $Dz$and $Rz$); $Qz_{2}$(ZnZntR-DNA complex); $Qz_{3}$(transcription initiation complex formed by $Qz_{2}$ and $Rz$); $Qz_{4}$(apo-ZntR-DNA complex); $Qz_{5}$(transcription initiation complex formed by $Qz_{4}$ and $Rz$); $w$(ZnuC);$Dw$(DNA of ZnuC); $Rw$(RNA polymerase for *znuC* transcription); $Mw$(mRNA of ZnuC); $Qw_{1}$(transcription initiation complex of ZnuC); $Qw_{2}$(Zn_4_Zur-DNA complex which can not further bind with $Rw$); $Tp$(free TPEN not bounded by zinc); $Tp_{1}$(zinc-bound TPEN).

## *Equations for Zur-DNA interaction*

$$\begin{matrix} \frac{dPy(t)}{dt}=-r_{3}Zn^{2}Py(t)+r_{4}Py_{1}(t) \\ \frac{dPy_{1}(t)}{dt}=r_{3}Zn^{2}Py(t)-r_{4}Py_{1}(t)-k_{1a}Dw(t)Py_{1}(t)+k_{-1}Qw_{2}(t) \\ \frac{dDw(t)}{dt}=-k_{1a}Dw(t)Py_{1}(t)+k_{-1}Qw_{2}(t) \\ \frac{dQw_{2}(t)}{dt}=k_{1a}Dw(t)Py_{1}(t)-k_{-1}Qw_{2}(t) \end{matrix}$$

By imposing all the derivatives to be 0, we get four steady state equations which contain only two independent equations. Therefore, in order to derive the steady state values, two conservation restraints need to be included:

$$Py(t)+Py_{1}(t)+Qw_{2}(t)=Py_{tot}=25nM,Dw(t)+Qw_{2}(t)=D_{0}=1nM$$

By solving the two independent steady state equations with the above conservation restraints, we can get the steady state concentration value of Zn_4_Zur-DNA complex (i.e., $Qw_{2}^{s}$) as a function of parameter $Zn$as follows:

$$\begin{matrix} Qw_{2}^{s}=f(Zn) \\ =Py_{tot}+\frac{Zn^{2}D_{0}k_{1a}r_{3}+Zn^{2}k_{-1}r_{3}-Zn^{2}Py_{tot}k_{1a}r_{3}+D_{0}k_{1a}r_{4}+2k_{-1}r_{4}-Py_{tot}k_{1a}r_{4}}{2(Zn^{2}k_{1a}r_{3}+k_{1a}r_{4})} \\ -\frac{\sqrt{4Zn^{2}k_{-1}k_{1a}r_{3}Py_{tot}(Zn^{2}r_{3}+r_{4})+(Zn^{2}D_{0}k_{1a}r_{3}+Zn^{2}k_{-1}r_{3}-Zn^{2}Py_{tot}k_{1a}r_{3}+k_{-1}r_{4})^{2}}}{2(Zn^{2}k_{1a}r_{3}+k_{1a}r_{4})} \\ +\frac{k_{-1}r_{4}^{2}-r_{4}\sqrt{4Zn^{2}k_{-1}k_{1a}r_{3}Py_{tot}(Zn^{2}r_{3}+r_{4})+(Zn^{2}D_{0}k_{1a}r_{3}+Zn^{2}k_{-1}r_{3}-Zn^{2}Py_{tot}k_{1a}r_{3}+k_{-1}r_{4})^{2}}}{2Zn^{2}r_{3}(Zn^{2}k_{1a}r_{3}+k_{1a}r_{4})} \end{matrix}$$

## *Equations for Zur transcription assay*

*(1) Equations for preliminary equilibrium of reactants before NTPs were added*

$$\begin{matrix} \frac{dPy(t)}{dt}=-r_{3}Zn^{2}Py(t)+r_{4}Py_{1}(t) \\ \frac{dPy_{1}(t)}{dt}=r_{3}Zn^{2}Py(t)-r_{4}Py_{1}(t)-k_{1a}Dw(t)Py_{1}(t)+k_{-1}Qw_{2}(t) \\ \frac{dDw(t)}{dt}=-k_{1a}Dw(t)Py_{1}(t)+k_{-1}Qw_{2}(t)+k_{-2}Qw_{1}(t)-k_{2}Dw(t)Rw(t) \\ \frac{dRw(t)}{dt}=k_{-2}Qw_{1}(t)-k_{2}Dw(t)Rw(t) \\ \frac{dQw_{1}(t)}{dt}=-k_{-2}Qw_{1}(t)+k_{2}Dw(t)Rw(t) \\ \frac{dQw_{2}(t)}{dt}=k_{1a}Dw(t)Py_{1}(t)-k_{-1}Qw_{2}(t) \end{matrix}$$

*(2) Equations for Zur run-off transcription after NTPs were added* $\begin{matrix} \frac{dPy(t)}{dt}=-r_{3}Zn^{2}Py(t)+r_{4}Py_{1}(t) \\ \frac{dPy_{1}(t)}{dt}=r_{3}Zn^{2}Py(t)-r_{4}Py_{1}(t)-k_{1a}Dw(t)Py_{1}(t)+k_{-1}Qw_{2}(t) \\ \frac{dDw(t)}{dt}=-k_{1a}Dw(t)Py_{1}(t)+k_{-1}Qw_{2}(t)+k_{3}Qw_{1}(t)+k_{-2}Qw_{1}(t)-k_{2}Dw(t)Rw(t) \\ \frac{dRw(t)}{dt}=k_{3}Qw_{1}(t)+k_{-2}Qw_{1}(t)-k_{2}Dw(t)Rw(t) \\ \frac{dQw_{1}(t)}{dt}=-k_{3}Qw_{1}(t)-k_{-2}Qw_{1}(t)+k_{2}Dw(t)Rw(t) \\ \frac{dQw_{2}(t)}{dt}=k_{1a}Dw(t)Py_{1}(t)-k_{-1}Qw_{2}(t) \\ \frac{dMw(t)}{dt}=k_{3}Qw_{1}(t) \end{matrix}$

***Equations for ZntR transcription assay (I)***

*(1) Equations for preliminary equilibrium of reactants before NTPs were added*

$$\begin{matrix} \frac{dPx(t)}{dt}=-r_{1}ZnPx(t)+r_{2}Px_{1}(t)-k_{1b}Dz(t)Px(t)+k_{-1}Qz_{4}(t) \\ \frac{dPx_{1}(t)}{dt}=r_{1}ZnPx(t)-r_{2}Px_{1}(t)-k_{1}Dz(t)Px_{1}(t)+k_{-1}Qz_{2}(t) \\ \frac{dDz(t)}{dt}=-k_{1b}Dz(t)Px(t)-k_{1}Dz(t)Px_{1}(t)+k_{-1}Qz_{2}(t)+k_{-2}Qz_{1}(t)+k_{-1}Qz_{4}(t)-k_{2a}Dz(t)Rz(t) \\ \frac{dRz(t)}{dt}=k_{-2}Qz_{1}(t)+k_{-2}Qz_{3}(t)+k_{-2}Qz_{5}(t)-k_{2a}Dz(t)Rz(t)-k_{2b}Qz_{4}(t)Rz(t)-k_{2c}Qz_{2}(t)Rz(t) \\ \frac{dQz_{1}(t)}{dt}=-k_{-2}Qz_{1}(t)+k_{2a}Dz(t)Rz(t) \\ \frac{dQz_{2}(t)}{dt}=k_{1}Dz(t)Px_{1}(t)-k_{-1}Qz_{2}(t)+k_{-2}Qz_{3}(t)-k_{2c}Qz_{2}(t)Rz(t) \\ \frac{dQz_{3}(t)}{dt}=-k_{-2}Qz_{3}(t)+k_{2c}Qz_{2}(t)Rz(t) \\ \frac{dQz_{4}(t)}{dt}=k_{1b}Dz(t)Px(t)-k_{-1}Qz_{4}(t)+k_{-2}Qz_{5}(t)-k_{2b}Qz_{4}(t)Rz(t) \\ \frac{dQz_{5}(t)}{dt}=-k_{-2}Qz_{5}(t)+k_{2b}Qz_{4}(t)Rz(t) \end{matrix}$$

*(2) Equations for ZntR run-off transcription (I) after NTPs were added*

***Equations for ZntR transcription assay (II) - including TPEN reaction***

*(1) Equations for preliminary equilibrium of reactants before NTPs were added*

*(2) Equations for ZntR run-off transcription (II) after NTPs were added*
