# Supplementary material for: Simulating in vitro transcriptional response of zinc homeostasis system in Escherichia coli
Source: BMC Syst Biol. 2008 Oct 24;2:89. doi: 10.1186/1752-0509-2-89 (PMC2611976; doi:10.1186/1752-0509-2-89)

***Transient curves of simulated Zur transcription assay for parameter $Zn=10^{-6}nM$:***


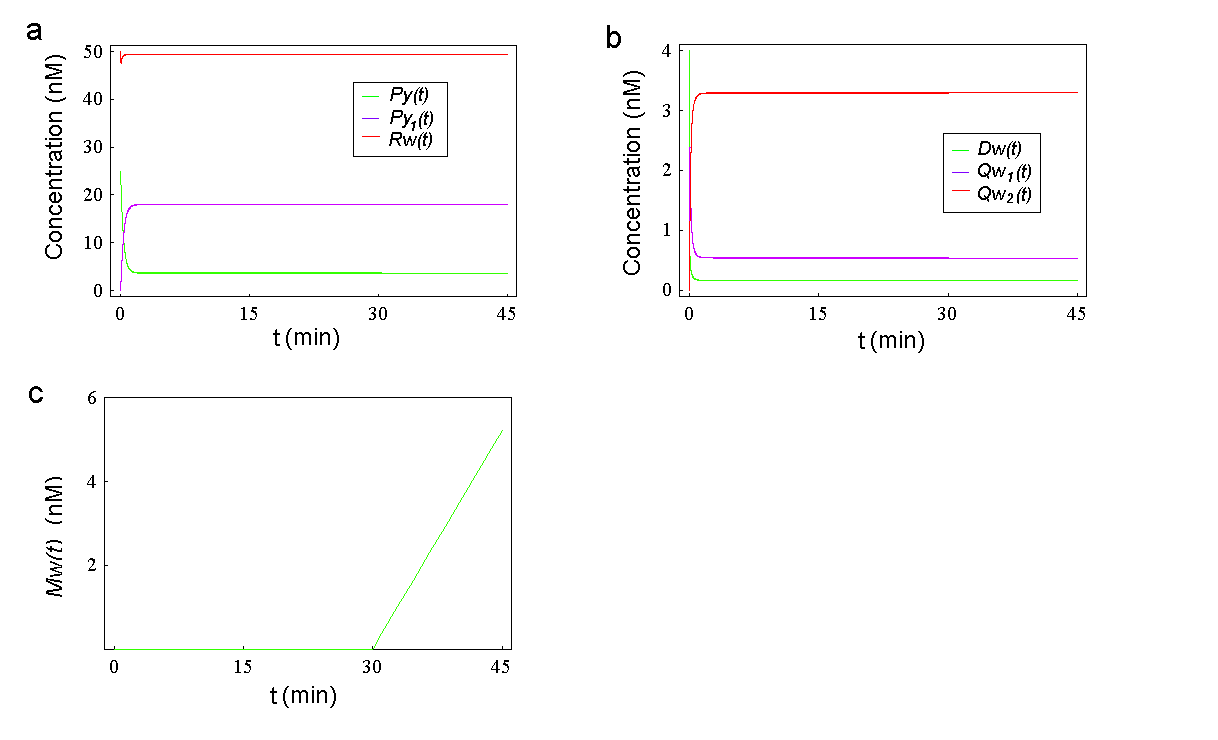


***Transient curves of simulated Zur transcription assay for parameter $Zn=10^{-7}nM$:***


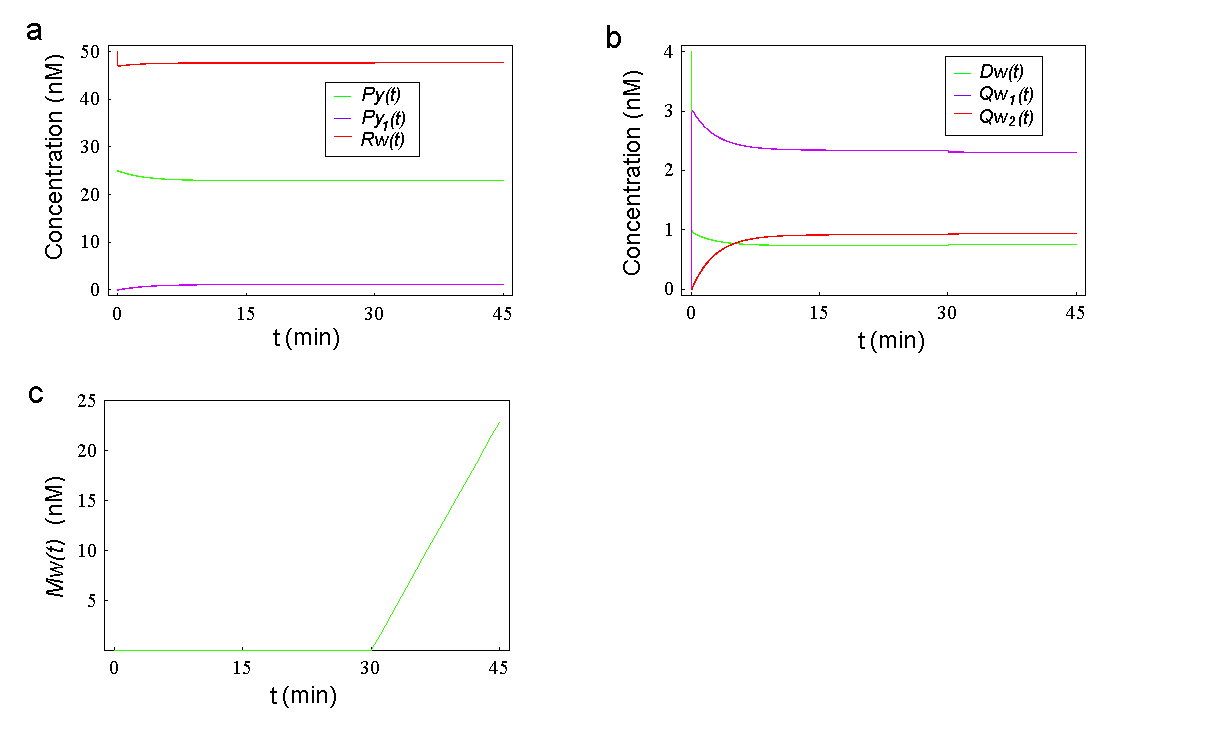


***Transient curves of simulated ZntR transcription assay (I) for*** ***parameter $Zn=10^{-6}nM$:***


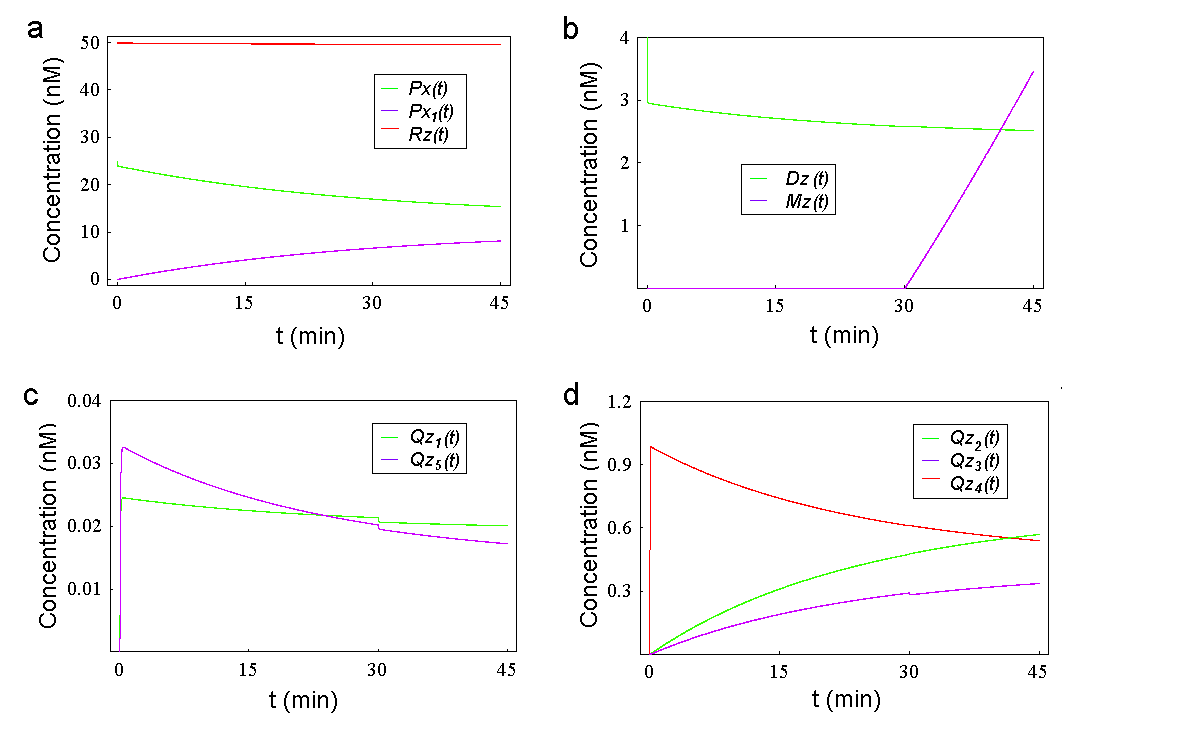


***Transient curves of simulated ZntR transcription assay (I) for*** ***parameter $Zn=10^{-7}nM$:***


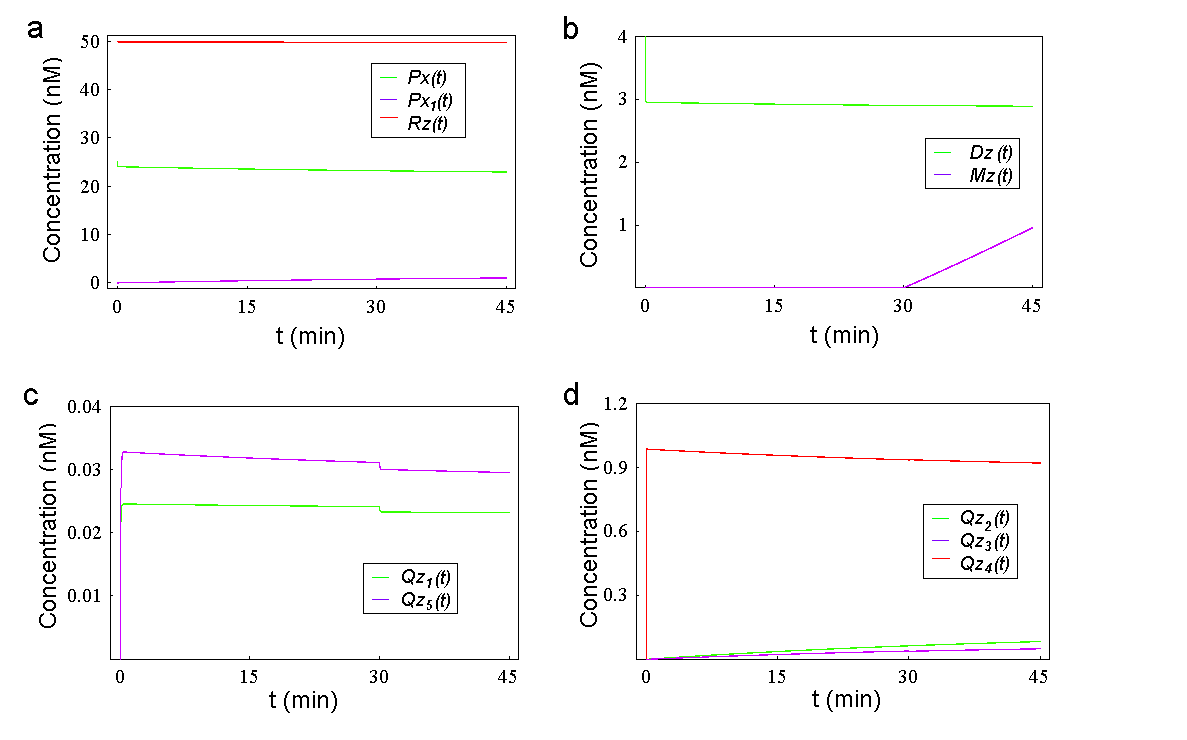

Supplement: Additional file 2 — MoreTransientCurves.doc. This additional file describes the simulated transient curves of Zur and ZntR transcription assays for parameter Zn = 10-6nM, 10-7nM. [file 1752-0509-2-89-S2.doc]
